# First report of pharmaceuticals and personal care products in two tropical rivers of southwestern India

**DOI:** 10.1007/s10661-020-08480-2

**Published:** 2020-07-17

**Authors:** Derrick Ian Joshua, Yerabham Praveenkumarreddy, Valiparambil Prabhakaranunni Prabhasankar, Andrea Petula D’Souza, Nobuyoshi Yamashita, Keshava Balakrishna

**Affiliations:** 1 Department of Civil Engineering, Manipal Institute of Technology, Manipal Academy of Higher Education, Manipal, 576104 India; 2 Department of Civil Engineering, Christ College of Engineering, Irinjalakuda, Kerala 680125 India; 3 Department of Geography and Environmental Management, Faculty of Environment, University of Waterloo, 200 University Avenue West, Waterloo, ON N2L 3G1 Canada; 4 National Institute of Advanced Industrial Science and Technology (AIST), 16-1 Onogawa, Tsukuba, Ibaraki 305-8569 Japan

**Keywords:** Pharmaceuticals, Sulfamethoxazole, Nethravati river, Swarna river, Seasonal variations

## Abstract

The occurrence of selected pharmaceuticals (trimethoprim, sulfamethoxazole, chloramphenicol, bezafibrate, ceftriaxone, and naproxen) in two west-flowing tropical rivers (Swarna and Nethravati) of southwestern India is reported for the first time. Water samples were collected during the monsoon and post-monsoon seasons from river water end members and further downstream up to their confluence with the adjacent Arabian Sea. Samples were analyzed using HPLC–MS/MS. Results revealed that there were no significant seasonal variations in concentrations of target analytes in both the rivers. Of the total number of samples analyzed (*n* = 24), trimethoprim was detected in 100% of the samples, whereas sulfamethoxazole (SMX), chloramphenicol (CAP), ceftriaxone (CTX), and naproxen (NPX) were detected in between 91 and 58% of the samples. Bezafibrate (BZF) was not detected in the samples. Nethravathi river showed higher concentrations of pharmaceuticals than the Swarna river which may be attributed to comparatively larger human population in the basin. Possible impacts of PPCPs on aquatic life offer further scope for study.

## Introduction

The term “pharmaceuticals” includes a wide range of prescription and non-prescription drugs for humans and animal use (Daughton and Ternes 1999). Significant portions of these drugs pass through the human body unchanged or metabolize to different biologically active substances that are ultimately released into the water bodies (Kümmerer 2009). The pathways for movement of these emerging pollutants into the water bodies are often from sewage treatment plants, aquaculture, and agricultural effluents. Once in the aquatic environment, pathogens are known to ingest it in low doses and acquire antibiotic resistance (Kummerer 2004).

Various studies done worldwide have reported the occurrence of PPCPs in surface, drinking and ground water, as well as soils and sediments, with concentrations ranging from ng L^−1^ to μg L^−1^ (Ternes et al. 2007; Moldovan 2006). A study made by the U.S. Geological Survey (USGS), sampling 139 streams from 30 States of the USA, reported that PPCPs were found in 80% of the streams (Kolpin et al. 2002). Recently, PPCPs are reported from rivers in China (Feng et al. 2020; Jia et al. 2018), Sri Lanka (Guruge et al. 2019), South Africa (Matongo et al. 2015), and United Kingdom (White et al. 2019). Danner et al. (2019) reviewed the antibiotic pollution in surface waters of Africa, Americas, Asia-Pacific, and Europe.

Only a handful of studies on PPCPs in wastewater and river waters are reported from India which is reviewed by Balakrishna et al. (2017) and Philip et al. (2018). The source of PPCPs to the rivers of India is primarily from wastewater (Mutiyar and Mittal 2014). Subedi et al. (2017) reported that only 31% of the total sewage generated in India is treated. Diwan et al. (2010) detected eight antibiotics in hospital effluents of Ujjain district which were in trace concentrations. Larsson et al. (2007) reported extremely high concentrations of PPCPs (31 ppm of ciprofloxacin) in a treatment plant that received effluents from 90 pharmaceutical industries in Patancheru, near Hyderabad. They also predicted that the effluents could be toxic to the microorganisms in the aquatic ecosystem where the effluents are ultimately discharged. Shanmugam et al. (2014) investigated five non-steroidal anti-inflammatory drugs in Kaveri, Vellar, and Tamiraparani rivers in Tamil Nadu. They observed elevated concentrations of acetylsalicylic acid in these rivers which could be directly discharged to these rivers from untreated sewage. More recently, pharmaceuticals were reported from Ganges, Brahmaputra and Arkavathi rivers in India (Sharma et al. 2019; Kumar et al 2019; Gopal et al. 2020) 

## Materials and methods

### Study area and sample collection

**Fig. 1:**
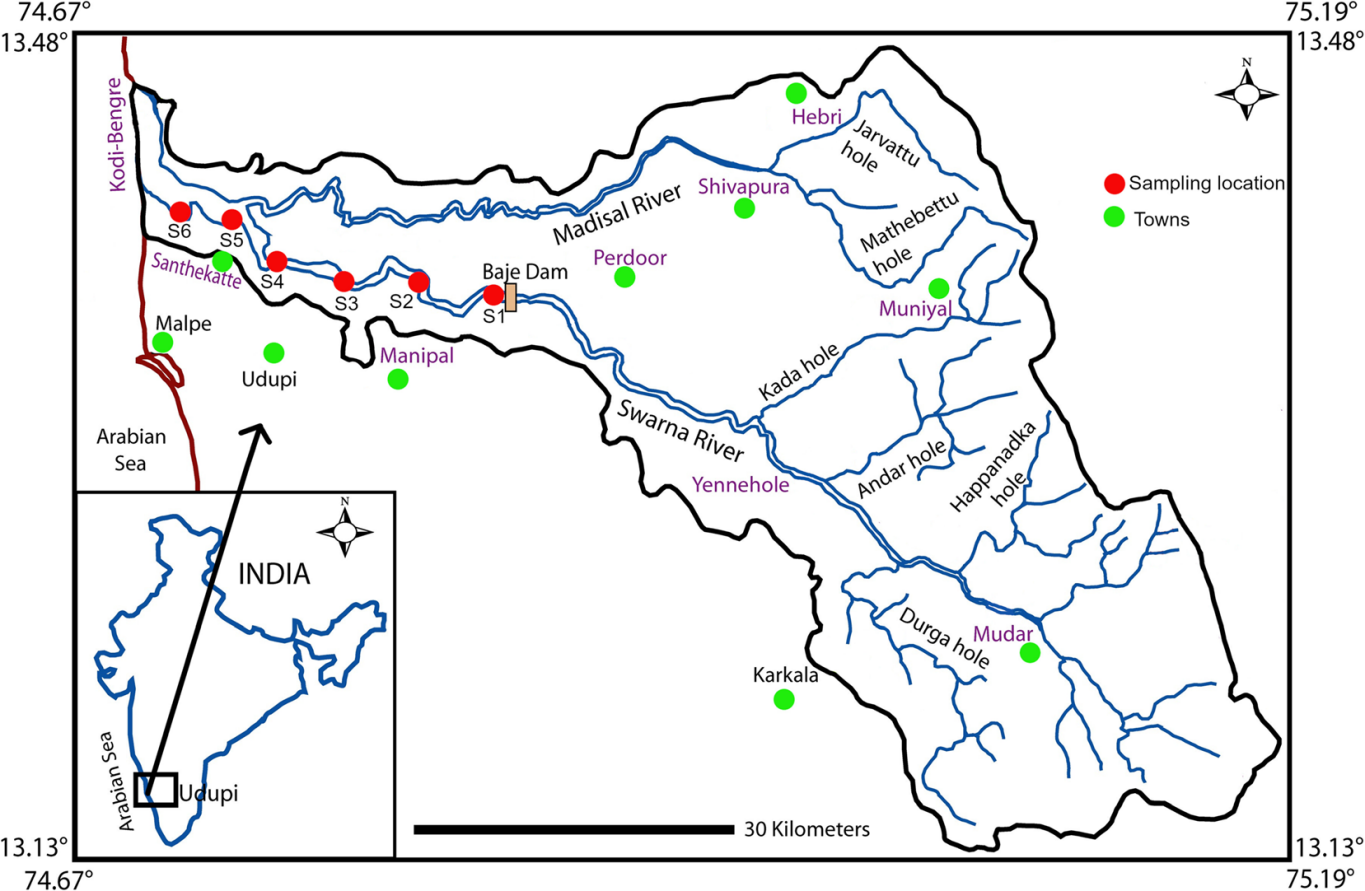
Location map showing sampling stations in R. Swarna

River (R.) Swarna is perennial, originating in the Western Ghats and flows along the southwest coast in India with an average discharge of 54 m^3^ s^−1^ (Tripti et al. 2013). The river forms a major source of drinking water for the town of Udupi. The river joins the Arabian Sea at Kodibengere (Fig. 1).

**Fig. 2:**
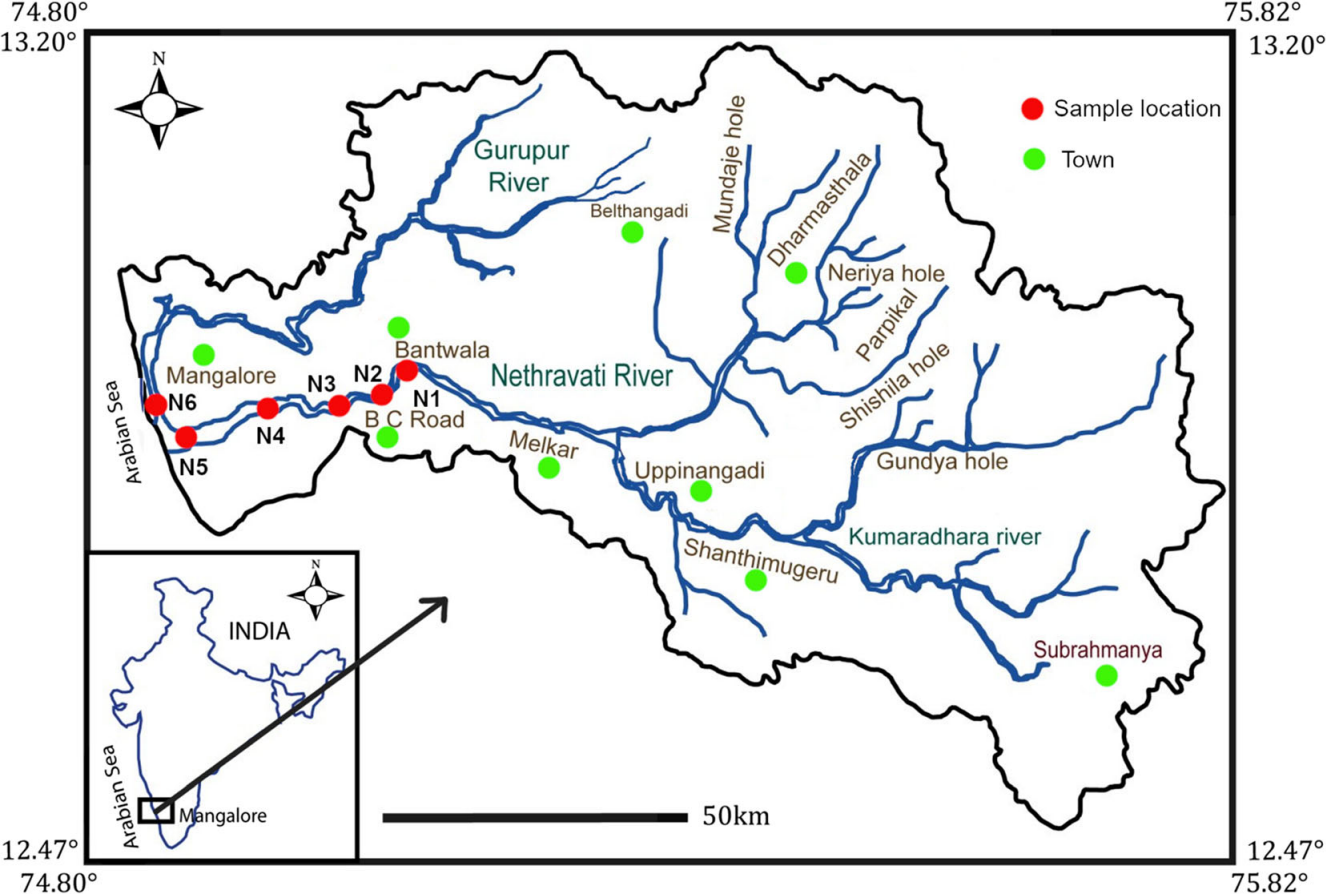
Location map showing sampling stations in R. Netravathi

River (R.) Netravathi is west-flowing, originating at Gangamoola in the Western Ghats, and flows towards the Arabian Sea for 147 km in the southwest direction (Fig. 2). The river joins the Arabian Sea near Mangalore. It drains a total area of 3657 km^2^ with a discharge of 336 m^3^ s^−1^ (Higgins et al. 2018). This river forms a major source of drinking water to Mangalore city. The mouth of R. Nethravathi is 67 km south of the mouth of R. Swarna

Six pharmaceuticals were chosen for this study, viz. trimethoprim (TMP), sulfamethoxazole (SMX), chloramphenicol (CAP), bezafibrate (BZF), ceftriaxone (CTX), and naproxen (NPX). Water samples (*n* = 24) were collected from six stations during September 2012 and November 2012, which are representative of monsoon and post-monsoon seasons (S1 (Baje dam) to S6 (Kodibengre), R. Swarna) & N1 (BC road) to N6 (Bolar), R. Netravathi). Water samples were collected from the center of the road bridge or using boats, in polypropylene bottles (500 mL) and stored at 253.15 K (− 20 °C) before extraction. The first sample was collected from the river end members (salinity = 0 PSU) of both the rivers. The rest of the samples were collected further downstream until the confluence of the rivers with the Arabian Sea.

### Chemicals and standards

For solid-phase extraction (SPE), Oasis® hydrophilic-lipophilic balance (HLB) cartridges **(**6 cc, 200 mg) (Waters, Milford MA) were used. Disodium ethylenediaminetetraacetic acid (Na_2_EDTA), high-performance liquid chromatography (HPLC) grade methanol, standards for ampicillin (AMP), bezafibrate (BZF), naproxen (NPX), chloramphenicol (CAP) along with isotope-labeled internal standards were purchased from WPCI Ltd. (Tokyo, Japan). Standards of erythromycin (ERY), trimethoprim (TMP), and their isotope-labeled internal standards were procured from Sigma-Aldrich (St. Louis, MO, USA). The sodium salt of CTX was purchased from Fluka (Buchs, Switzerland). Methanol was used to prepare the individual pharmaceutical compounds, and a mixed standard of pharmaceutical compounds was prepared using 25% methanol as per procedure reported in Gulkowska et al. (2007). All standards had a purity of ≥ 95%.

### Sample extraction

Detailed sample extraction process is reported elsewhere by these authors (Prabhasankar et al. 2016). Briefly, 5 mL of methanol and 5 mL of deionized (Milli Q) water were used for the preconditioning of the HLB sorbent. The cartridges were loaded with the sample at a flow rate of 3 mL min^−1^. The cartridges were washed and centrifuged to remove the retained water. Target analytes were eluted with methanol and the eluted solution was evaporated to near dryness at 303.15 K (30 °C) using nitrogen gas. The sample was reconstituted to 1 mL using 25% methanol and preserved for LC–MS/MS analysis.

### Instrument analysis

HPLC was used to analyze the concentration of PPCPs in the samples with tandem mass spectrometry (HPLC-MS-MS). Agilent HP1100 liquid chromatography (Agilent, Palo Alto, CA) was used to separate the analytes, and the concentration was quantified by Applied Biosystems API, 2000^TM^ triple quadrupole tandem mass spectrometer. The mobile phase used for the analysis was 2 mM ammonium acetate. Analysis was done at AIST, Tsukuba, Japan.

### Quality assurance and quality control

Field blanks and method blanks were prepared during the sample collection along with deionized water. Limit of quantification (LOQ) was determined based on signal to noise ratio of 10:1 or greater. Only those target analytes whose concentrations were observed to be in this ratio and above were considered. In the cartridge, to reduce the chances of the possible degradation of the target compounds due to time, different sample matrices (river water and deionized water) were used and processed at different temperatures (temperature stability analysis). Standards were used to spike these two matrices and were then stored under two conditions, room temperature and at 277.15 K (4 °C). Recovery tests were conducted on the same day and after one day followed by 3rd, 9th, and 30 days of storage. Except AMP, negligible variation was seen with respect to other target compounds in time, temperature, and matrix. This test confirmed that in comparison to deionized water, AMP is more stable in river water. Zero percent recovery was observed for AMP after 30 days for both deionized and river water. This could be the possible reason for the low concentrations of AMP in the WTP samples.

A total of 100 μL volume (100 ng mL^−1^ concentration) isotope-labeled internal standards of the respective target analytes was spiked to deionized water to estimate the recovery. The target analyte recovery percentages were between 70 and 100% for SMX, TMP, and CAP. Recovery percentages of ERY and AMP were < 50% (Prabhasankar et al. 2016). Quantification of target analytes in the samples was based on external calibration curves that were constructed for six concentrations (1, 5, 10, 20, 50, and 100 ng L^−1^).

## Results and discussion

**Fig. 3:**
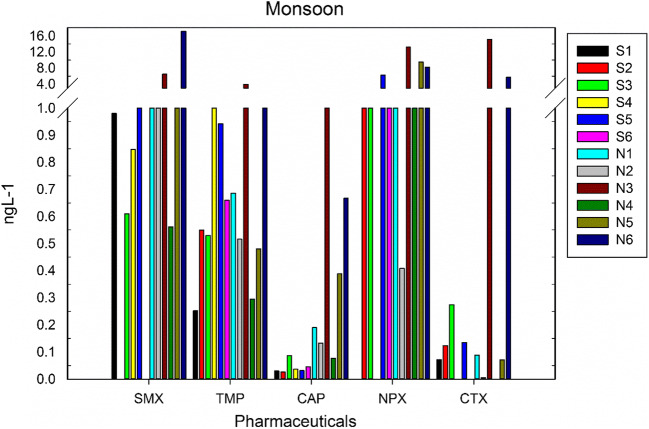
Concentration of pharmaceutical compounds in the R. Swarna (S1–S6) and R. Nethravathi (N1–N6) during monsoon season

**Fig. 4:**
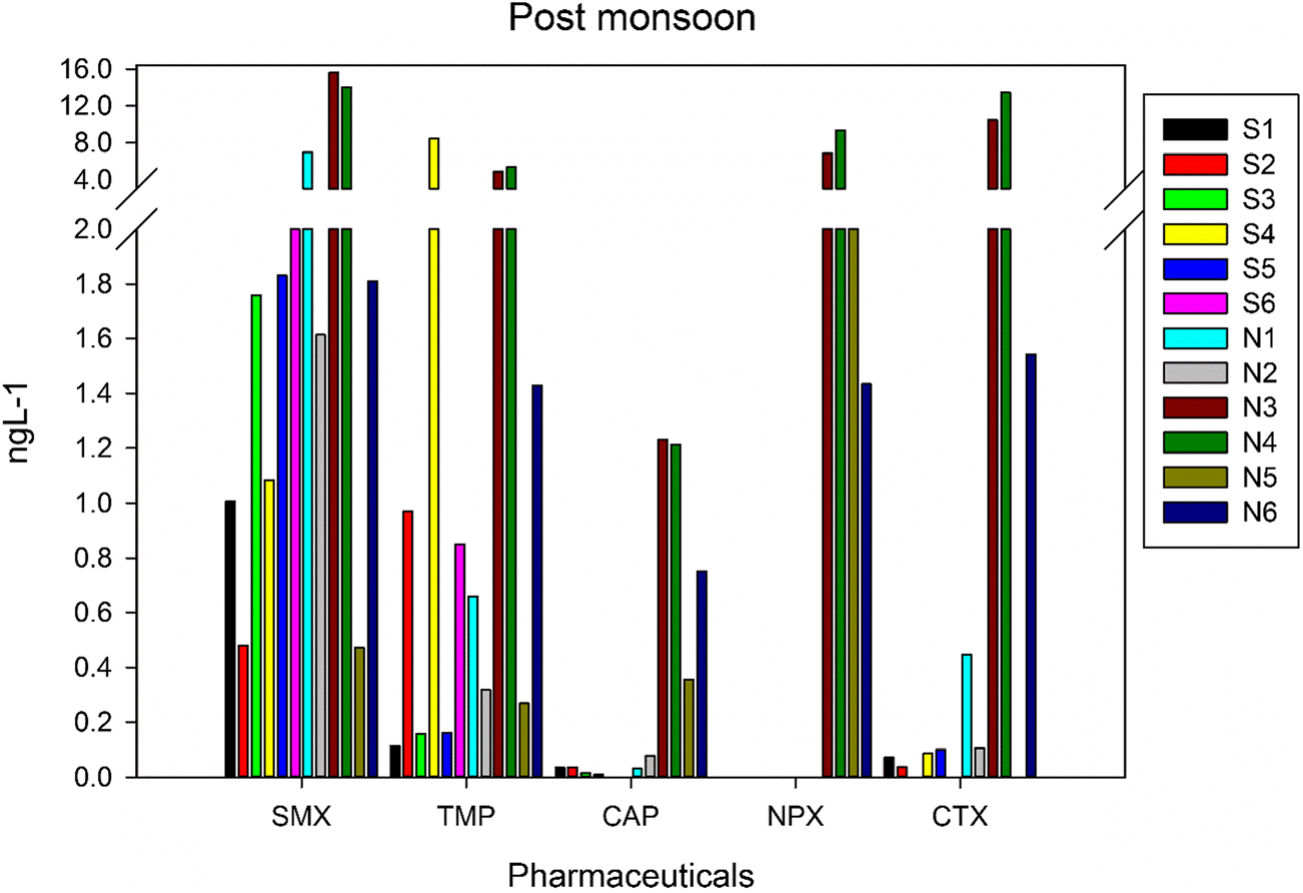
Concentration of pharmaceutical compounds in the R. Swarna (S1–S6) and R. Nethravathi (N1–N6) during post-monsoon season

To our knowledge, this is the first study to report pharmaceuticals in waters of R. Swarna and R. Netravathi. The monsoon samples from R. Swarna showed the presence of SMX at four stations and TMP at six stations (Fig. 3, Table 1). Corresponding samples of post-monsoon showed the presence of SMX and TMP at all the stations (Fig. 4, Table 2). The highest concentration of both SMX and TMP was in the post-monsoon season compared to monsoon season. This could be because of the amplification of these compounds due to the low discharge during the post-monsoon season. In both the seasons, in station S1 (Baje village) which is the source of drinking water for Udupi city, SMX was present at ~ 1 ng L^−1^. This concentration was fluctuating in the river from not detectable to ~ 1 ng L^−1^ throughout its course downstream in monsoon and fluctuating between 0.5 and 2 ng L^−1^ in post-monsoon. TMP was detected with low concentration (< 1 ng L^−1^) in monsoon and post monsoon seasons except in station S4 (Kalyanpur bridge), where the concentrations were much higher in both the seasons (1 ng L^−1^ in monsoon and 8 ng L^−1^ in post-monsoon). It is observed that many vehicles dump their garbage in this bridge, which could be a possible reason for higher concentration. CAP and CTX were detected in 83% of the samples in low concentrations (< 0.1 ng L^−1^ for CAP and < 0.3 ng L^−1^ for CTX) in R. Swarna for both the seasons. This may be due to the low consumption patterns and less population density at the river basin. NPX was not detected in post-monsoon season, whereas it was detected in 67% of the samples in the monsoon season. The concentrations ranged from 1.5 to 6.2 ng L^−1^.

**Table 1:** Concentrations (ng L^−1^) of selected pharmaceuticals in the R. Swarna and R. Nethravati during the monsoon season

Sampling site	SMX	TMP	CAP	NPX	CTX
Swarna River					
S1—Baje Dam	0.98	0.25	0.03	0.00	0.07
S2—Herga	0.00	0.55	0.03	2.80	0.12
S3—Vinayak Temple	0.61	0.53	0.09	2.62	0.27
S4—Kalyanpur Bridge	0.85	1.07	0.04	0.00	0.00
S5—Kalyanpur	1.02	0.94	0.03	6.24	0.14
S6—Kodibengre	0.00	0.66	0.05	1.50	0.00
Netravathi River					
N1—BC Road	1.41	0.69	0.19	1.26	0.09
N2—Thumbe	1.20	0.52	0.13	0.41	0.01
N3—Ullal Drain	6.45	3.97	2.27	13.22	15.14
N4—Ullal Bridge	0.56	0.29	0.08	1.16	0.00
N5—N. Mouth	1.51	0.48	0.39	9.48	0.07
N6—Bolar	17.13	2.39	0.67	8.21	5.69

**Table 2:** Concentrations (ng L^−1^) of selected pharmaceuticals in the R. Swarna and R. Nethravati during the post-monsoon season

Sampling site	SMX	TMP	CAP	NPX	CTX
Swarna River					
S1—Baje Dam	1.01	0.11	0.04	0.00	0.07
S2—Herga	0.48	0.97	0.04	0.00	0.04
S3—Vinayak Temple	1.76	0.16	0.02	0.00	0.00
S4—Kalyanpur Bridge	1.08	8.42	0.01	0.00	0.09
S5—Kalyanpur	1.83	0.16	0.00	0.00	0.10
S6—Kodibengre	2.00	0.85	0.00	0.00	0.00
Netravathi River					
N1—BC Road	6.96	0.66	0.03	0.00	0.45
N2—Thumbe	1.61	0.32	0.08	0.00	0.11
N3—Ullal Drain	15.59	4.87	1.23	6.87	10.45
N4—Ullal Bridge	14.05	5.35	1.21	9.32	13.45
N5—N. Mouth	0.47	0.27	0.36	2.36	0.00
N6—Bolar	1.81	1.43	0.75	1.43	1.54

In R. Netravathi, SMX and TMP were detected in all the stations. During monsoon, SMX concentrations ranged from 1.4 to 17 ng L^−1^ along the course of the river (Fig. 3, Table 1). SMX concentration in the post-monsoon at Thumbey village (N2) (drinking water source for Mangalore city) showed a concentration of 1.61 ng L^−1^ and increased by an order of magnitude at station N3 (Ullal drain; 15.6 ng L^−1^) (Fig. 4, Table 2). This increase may be attributed to the point source pollution contributed by the sewage from Mangalore city discharged into the R. Netravathi. TMP concentrations ranged from 0.3 to 4 ng L^−1^ in the monsoon, with the highest concentration near the point source at Ullal drain. In the post-monsoon too, high concentrations were observed at Ullal bridge and Ullal drain (~ 5 ng L^−1^) confirming the influence of the point source from the city’s wastewater outlet. CAP was detected in all the samples during the monsoon and post-monsoon seasons. The highest concentrations were observed in Ullal drain and Ullal bridge samples in both the seasons (1.2–2.3). NPX was detected in 83% of the stations. The concentrations in monsoon ranged from 0.4 to 13 ng L^−1^ and 0 to 9.32 ng L^−1^ in post-monsoon seasons. While the drinking water source for Mangalore city detected the lowest concentrations, the higher concentrations were detected in the downstream side, at Ullal drain, Ullal bridge, Nethravati mouth, and Bolar. CTX was detected in 83% of the stations.

The two rivers were compared for the frequency of occurrence of pharmaceuticals, and it was observed that R. Netravathi had a higher frequency of occurrence of SMX (100%) when compared to 83% for R. Swarna (Tables 1 and 2). TMP was found in equal numbers of samples in both the rivers. Similarly, NPX and CTX were found in 83%, CAP was found in 100% of R. Netravathi samples.

Of the total number of samples analyzed (*n* = 24), SMX was found in 81% of the samples followed by TMP in 100%, NPX in 33%, CAP in 83%, and CTX in 66% of the samples. A review of antibiotic concentrations in the global aquatic environment by Kümmerer (2009) shows that SMX concentration ranged from 52 to 1900 ng L^−1^ and TMP from 12 to 170 ng L^−1^. However, the concentration of corresponding antibiotics in this study was much lower, ranging from 0.47 to 17.13 ng L^−1^ for SMX and 0.27 to 5.35 ng L^−1^ for TMP. R. Netravathi shows NPX concentration ranging from 0 to 13.22 ng L^−1^ alongside NPX concentrations in Qiantang River, China, which showed concentrations below 15 ng L^−1^ (Chen et al. 2012). CAP was reported in German surface waters at 60 ng L^−1^ (Hirsch et al. 1999) compared to 1 ng L^−1^ in R. Netravathi. Exposure to low levels of pharmaceuticals in rivers (up to 100 ng L^−1^) is generally not considered a human health risk (Fick et al. 2009). However, this concentration is sufficient to develop antibiotic resistance in the bacterial populations due to selective pressure. This is supported by the recent studies done by Praveenkumarreddy et al. 2020, where they found antibiotic resistant genes in the wastewater effluents of the treatment plants in southern India. In addition, the effect of low levels of pharmaceuticals and its toxicity on aquatic life forms requires further study. Sui et al. (2012) have proposed a priority list of pharmaceuticals to be monitored in China, based on consumption, efficiency of WTPs in treating it, and its effect on ecology. A similar compilation in India is needed which requires a nationwide survey of major rivers, groundwater, and treated wastewater. This would enable policy changes that could ensure controlled release of pharmaceuticals to the water bodies. Furthermore, there is an immediate need of cost-effective treatment of PPCPs to ensure its 100% removal in WTPs. A combination of biological treatment and ozonation can be a promising technique for the removal of PPCPs.

## Conclusion

This is the first study reporting the occurrence of six pharmaceuticals (trimethoprim, sulfamethoxazole, chloramphenicol), bezafibrate, ceftriaxone and naproxen), in two small tropical rivers, Swarna, and Netravathi flowing across the southwestern India. These rivers are also the primary drinking water source of two major coastal cities Mangalore and Udupi in southwestern coast of India. The occurrence of the pharmaceuticals is observed in a higher frequency and higher concentrations in R. Netravathi compared to R. Swarna. In R. Netravathi, the increase in the concentrations of the pharmaceuticals could be due to the point source pollution contributed by domestic sewage discharge. In the post-monsoon season, in both the rivers, the concentrations are higher than the monsoon due to the low discharge of water and the amplification of concentrations due to sewage discharged from municipal outlets. In the monsoon season, R. Swarna showed relatively higher presence of sulfamethoxazole, trimethoprim, naproxen compared to chloramphenicol and ceftriaxone. Similar behavior was observed in the post-monsoon season, though naproxen was not detected. Long-term exposure of the pharmaceuticals may affect the aquatic vertebrates especially the gene responses and hormonal levels. Pharmaceuticals present in these concentrations (below 100 ng L^−1^) are believed to be of no human health risk. However, their impact on bacterial populations, especially related to the development of antibiotic resistance due to selective pressure, is a concern that should be addressed in the future.
